# Management of venous thromboembolism: an update

**DOI:** 10.1186/s12959-016-0107-z

**Published:** 2016-10-04

**Authors:** Siavash Piran, Sam Schulman

**Affiliations:** Department of Medicine, Division of Hematology and Thromboembolism, and Thrombosis and Atherosclerosis Research Institute, McMaster University, Hamilton, ON L8L 2X2 Canada

**Keywords:** Venous thromboembolism, Anticoagulation, Direct oral anticoagulants, Vitamin K antagonists

## Abstract

Venous thromboembolism (VTE), which constitutes pulmonary embolism and deep vein thrombosis, is a common disorder associated with significant morbidity and mortality. Landmark trials have shown that direct oral anticoagulants (DOACs) are as effective as conventional anticoagulation with vitamin K antagonists (VKA) in prevention of VTE recurrence and associated with less bleeding. This has paved the way for the recently published guidelines to change their recommendations in favor of DOACs in acute and long-term treatment of VTE in patients without cancer. The recommended treatment of VTE in cancer patients remains low-molecular-weight heparin. The initial management of pulmonary embolism (PE) should be directed based on established risk stratification scores. Thrombolysis is an available option for patients with hemodynamically significant PE. Recent data suggests that low-risk patients with acute PE can safely be treated as outpatients if home circumstances are adequate. There is lack of support for use of inferior vena cava filters in patients on anticoagulation. This review describes the acute, long-term, and extended treatment of VTE and recent evidence on the management of sub-segmental PE.

## Background

Venous thromboembolism (VTE), which includes deep vein thrombosis (DVT) and pulmonary embolism (PE), is one of the most common cardiovascular diseases occurring for the first time in about 1 in 1000 people [1, 2]. Its incidence rises with increasing age, for example to about 5 per 1000 people among those over 70 years of age [3]. VTE is associated with significant morbidity and mortality with the 30-day mortality rate in the absence of treatment of about 3 % for DVT and 31 % for PE [4]. The long-term complications of VTE are post-thrombotic syndrome (PTS), which occurs in 20 to 50 % of patients with DVT [5], and chronic thromboembolic pulmonary hypertension (CTEPH), which occurs in 2 to 4 % of patients with PE [6]. Patients with CTEPH have progressive dyspnea and exercise intolerance and those with PTS have chronic leg pain and swelling, which in a minority of patients can progress to development of venous ulcers. These conditions can significantly reduce the patient’s quality of life. Furthermore, the management VTE is associated with substantial health care costs for not only the initial hospitalization but also for hospital re-admissions [7, 8]. Therefore, VTE is associated with significant morbidity and mortality.

### Initial management

The initial management of patients with a PE should be based on risk stratification of the patient into low, intermediate, or high risk for 30-day mortality based on established risk scores such as pulmonary embolism severity index (PESI) or its simplified version (simplified PESI) [9, 10]. Low risk patients, who are hemodynamically stable, can be treated as outpatients if home circumstances are adequate [11, 12]. At the other extreme, patients with acute PE and hypotension or patients with DVT-associated phlegmasia of the lower leg should be considered for treatment with thrombolytic agents [13, 14].

### Oral anticoagulants

Anticoagulants are the mainstay treatment of VTE and are given in three phases of acute, long-term (in the first 3 months), and extended treatment [14]. For many years initial treatment was started with a parenteral anticoagulant, for example low-molecular-weight heparin (LMWH), overlapping with a vitamin K antagonist (VKA), such as warfarin. The combination was continued for at least 5 days until the achievement of therapeutic anticoagulation with international normalized ratio of 2 to 3 [14]. Although conventional therapy with VKAs is effective and safe, it has some limitations including delayed onset, need for parental daily injections, and interactions with dietary vitamin K and numerous drugs. Over the past 5 years, 4 direct oral anticoagulants (DOACs) have been approved for acute and long-term treatment of VTE [15–20]. The DOACs were compared with conventional therapy and found to be as effective in prevention of VTE recurrence and associated with less bleeding. The recently published American College of Chest Physicians (ACCP) guidelines have changed their recommendations in favor of DOACs in acute and long-term treatment of VTE in patients without cancer [21]. In patients with cancer associated VTE, the recommended anticoagulation remains LMWH over VKA [21].

The aim of this review is to (1) describe the initial management of patients with acute PE including the role of thrombolytic agents in hemodynamically unstable patients and at the other extreme outpatient management of low risk patients, (2) summarize the evidence on acute, long-term, and extended treatment of VTE comparing DOACs versus VKA, and (3) review the recent data on the management of sub-segmental PE and the lack of support for use of inferior vena cava filters in patients on anticoagulation.

## Review

### Acute and long-term treatment of venous thromboembolism

#### Thrombolytic and interventional treatment for acute venous thromboembolism

Anticoagulant therapy alone is recommended over thrombolysis for most patients with an acute DVT with exception for those with extensive iliofemoral or proximal DVT at high risk of limb ischemia [14, 21]. Thrombolytic therapy (systemic or catheter-directed) increase clot lysis and reduce the incidence of PTS compared to anticoagulation alone [22, 23]. However, this is at the expense of higher rate of major bleeding and no difference in rate of recurrent VTE or mortality [22–24]. Massive proximal DVT or iliofemoral thrombosis associated with limb-threatening ischemia or severe symptomatic swelling may be treated with thrombolysis. Thrombolysis can be considered only after objective diagnosis of the DVT and in a patient with low bleeding risk. The CaVenT trial randomized 209 patients with iliofemoral DVT to catheter directed therapy (CDT) versus anticoagulation. They found that the patients treated with CDT had significantly less PTS at 2 years compared with those treated with anticoagulation (41 versus 56 %) [22]. Another study randomized 32 patients with iliofemoral DVT to receive either CDT or systemic thrombolysis, followed by anticoagulation [25]. The patients who were treated with CDT had less reflux in both the deep and superficial veins and more patients had venous valvular competence preserved compared with patients who underwent systemic thrombolysis. A large, multicenter trial (the ATTRACT trial) is currently underway that randomizes patients to receive pharmaco-mechanical catheter-directed thrombolysis (PCDT) plus standard therapy with anticoagulation versus standard therapy alone [26]. It will investigate whether PCDT should be routinely utilized to prevent PTS in patients with symptomatic proximal DVT [26].

Systemic thrombolysis is a widely accepted treatment for PE in patients with persistent hypotension (e.g., systolic blood pressure <90 mmHg for 15 min) and not at high risk of bleeding [14, 21]. The use of thrombolytic therapy in intermediate risk patients with acute PE associated with right ventricle (RV) dysfunction is controversial. The RV dysfunction is confirmed by echocardiogram or computed tomography and a positive troponin I/T. The potential indication for thrombolysis in this group is based on evidence that patients with severe RV dysfunction have worse prognosis than those without RV dysfunction [27]. Three recently published trials have examined the role of systemic thrombolysis in intermediate risk patients [28–30]. In the Moderate Pulmonary Embolism Treated Thrombolysis (MOPETT) trial, 121 patients were randomly assigned to receive heparin (unfractionated or LMWH) alone or the combination of tissue type plasminogen activator (tPA) plus heparin [28]. Compared to the heparin group, treatment with tPA resulted in lower rates of pulmonary hypertension and significantly lower pulmonary artery systolic pressures at 28 months. The rates of bleeding, recurrent PE, and mortality was similar in both groups [28]. In another trial comparing the combination of LMWH plus an intravenous bolus of tenecteplase versus LMWH alone in intermediate risk PE patients, those treated with tenecteplase had fewer adverse outcomes and better functional capacity at 90 days [29]. In a large multicenter randomized trial (PEITHO), 1005 intermediate risk patients with PE were randomized to tenecteplase and heparin or to heparin therapy alone [30]. Thrombolysis therapy led to reduction in the primary composite outcome of death or cardiovascular collapse at seven days after randomization although it increased major bleeding (including intracranial bleeding) with no overall gained benefit from thrombolysis [30]. A meta-analysis of 16 trials comprising 2115 intermediate risk patients reported that 59 patients would need to be treated with thrombolysis to prevent one death, while a major bleeding occurs with every 18 patients treated [13]. Further studies are needed to identify subgroups of intermediate risk patients who will benefit from systemic thrombolytic therapy.

CDT may be used in patients with acute PE at increased risk of bleeding as a lower dose of a thrombolytic agent is infused directly into the pulmonary artery via a catheter [31]. CDT is also effective in lowering pulmonary arterial pressure and improving RV function [32]. In a randomized controlled trial of 59 patients with acute intermediate risk PE, ultrasound-assisted catheter-directed thrombolysis followed by heparin was compared to treatment with heparin alone [33]. At 24 h, CDT improved the hemodynamics compared to anticoagulation. At 90 days of follow-up, there was no difference in mortality or major bleeding between the two groups [33]. Most of the evidence is limited by small sample size and of low quality compared to the available evidence for systemic thrombolysis. Systemic thrombolysis is therefore currently recommended over CDT in patients with acute PE who are candidates for thrombolysis [21].

#### Outpatient treatment of venous thromboembolism

Home therapy is commonly employed for patients with an acute DVT in clinical practice with a few exceptions. Several randomized controlled trials and meta-analyses, which have compared home therapy with LMWH versus inpatient therapy with intravenous unfractionated heparin, suggest that outpatient therapy is safe and feasible in most patients with acute DVT [34–36]. Outpatient therapy should not be selected for those with massive symptomatic DVT, high risk of bleeding, or hemodynamic instability due to concurrent symptomatic PE [37].

The outpatient treatment of acute PE is suggested with grade 2B evidence in the most recent ACCP guidelines in low-risk patients with adequate home circumstances [21]. The decision for outpatient management should take into account the patient’s clinical condition, bleeding risk, their preference, and the available home support. Risk stratification scores such as PESI or simplified PESI may be utilized to identify low-risk patients without RV dysfunction who are potential candidates for short in-hospital stay or entirely outpatient management [11, 12, 38]. With the recent changed recommendations in favor of DOACs for acute and long-term VTE treatment, future research should focus on the safety and efficacy of DOACs in outpatient management of acute VTE.

#### Vitamin K antagonists versus direct oral anticoagulants

Four DOACs including dabigatran, rivaroxaban, apixaban, and edoxaban were compared with conventional therapy in the RE-COVER I and II, EINSTEIN-DVT and PE, AMPLIFY, and Hokusai-VTE trials, respectively [15–20]. The study design was double-blinded in all trials except for the ENSTEIN trials, which used a prospective, randomized, open-label, blinded end point evaluation design. The study designs and treat protocols are compared in Table 1. The study populations were similar in these trials. In the dabigatran and edoxaban trials, parental anticoagulation was added to both DOAC and conventional therapy arms, and after at least 5 days patients were switched to the DOAC. Therefore, in clinical practice, patients should be initiated on parenteral anticoagulation and either switched to dabigatran or edoxaban after 5 days or it should be overlapped with a vitamin K antagonist. In contrast, in the rivaroxaban and apixaban trials DOACs were started without the need for initial parental anticoagulation. The primary efficacy outcome was recurrent VTE or VTE-related mortality in all 6 trials. The primary safety outcome was either major bleeding or a composite of major and clinically relevant non-major bleeding (CRNMB). The efficacy and safety outcomes of these trials are listed in Table 2. All of the trials excluded patients with severe renal dysfunction, those with active bleeding or at high risk of bleeding, and patients already on therapeutic anticoagulation. A recent pooled analysis of these 6 trials reported that DOACs have similar efficacy as VKA in treatment of acute VTE and significantly lower risk of major bleeding than VKA [39]. Recurrent VTE occurred in 2 % of those given DOAC versus 2.2 % in patients that received VKA (relative risk [RR] 0.90; 95 % confidence interval [CI], 0.77 – 1.06) [39]. A 39 % reduction in risk of major bleeding was reported in DOAC recipients compared to those who received VKA therapy (RR 0.61; 95 % CI, 0.45 – 0.83). Compared with recipients of VKA therapy, intracranial bleeding, fatal bleeding, and CRNMB were significantly reduced in the DOAC group [39]. Given the better safety profile of DOACs with less major bleeding, similar efficacy in prevention of recurrent VTE, and the convenience of administration of DOACs, the recent ACCP guidelines suggested DOACs over VKA for the acute and long-term treatment of VTE in patients without cancer [21].

**Table 1:** Comparison of study design and treatment protocols of trials on DOACs Versus VKA for treatment of acute VTE

Trial name	RE-COVER	RE-COVER II	EINSTEIN-DVT	EINSTEIN-PE	AMPLIFY	Hokusai-VTE
Year of Publication [Ref]	2009 [15]	2014 [16]	2010 [17]	2012 [18]	2013 [19]	2013 [20]
Design	Double-blinded	Double-blinded	PROBE	PROBE	Double-blinded	Double-blinded
Number of Patients	2539	2589	3449	4832	5395	8292
Indication for Anticoagulation	Acute VTE	Acute VTE	Acute DVT	Acute PE	Acute VTE	Acute VTE
DOAC Treatment Protocol	Dabigatran 150 mg twice daily	Dabigatran 150 mg twice daily	Rivaroxaban 15 mg twice daily for 3 weeks; then 20 mg once daily	Rivaroxaban 15 mg twice daily for 3 weeks; then 20 mg once daily	Apixaban 10 mg twice daily for days; then 5 mg twice daily	Edoxaban 60 once daily; patients with CrCl 30–50 mL/min, body weight ≤60 kg, or receiving strong P-glycoprotein inhibitors: edoxaban 30 mg once daily
Non-inferiority Margin for Hazard Ratio	2.75	2.75	2.0	2.0	1.8	1.5
Need for initial Parenteral Anticoagulation	Yes	Yes	No	No	No	Yes
Duration of Therapy (months)	6	6	3, 6, or 12	3, 6, or 12	6	≤12
TTR (%)	60	57	58	63	61	64

*DOAC* direct oral anticoagulant, *DVT* deep vein thrombosis, *PE* pulmonary embolism, *PROBE* prospective, randomized, open-label, blinded end point, *TTR* time in therapeutic range for warfarin, *VKA* vitamin K antagonists, *VTE* venous thromboembolism, *CrCl* creatinine clearance

**Table 2:** Efficacy and safety outcomes for treatment of acute VTE: DOACs versus VKA

Trial Name [Ref]	RE-COVER [15]	RE-COVER II [16]	EINSTEIN-DVT [17]	EINSTEIN-PE [18]	AMPLIFY [19]	Hokusai-VTE [20]
Primary Efficacy Outcome DOAC vs VKA (%)	Recurrent symptomatic VTE or related death: 2.4 vs 2.1^a^	Recurrent symptomatic VTE or related mortality: 2.3 vs 2.2^a^	Recurrent symptomatic VTE: 2.1 vs 3.0^a^	Recurrent symptomatic VTE: 2.1 vs 1.8^a^	Recurrent symptomatic VTE or related mortality: 2.3 vs 2.7^a^	Recurrent symptomatic VTE or related mortality: 3.2 vs 3.5^a^
Primary Safety Outcome(s)	Major bleeding; Major or CRNM bleeding: Any bleeding	Major bleeding Major or CRNM bleeding: Any bleeding	Major or CRNM bleeding	Major or CRNM bleeding	Major bleeding	Major or CRNM bleeding
Major Bleeding DOAC vs VKA (%)	1.6 vs 1.9	1.2 vs 1.7	0.8 vs 1.2	1.1^a^ vs 2.2	0.6^a^ vs 1.8	1.4 vs 1.6
Major or CRNM Bleeding DOAC vs VKA (%)	5.6 vs 8.8	5.0 vs 7.9	8.1 vs 8.1	10.3 vs 11.4	4.3^a^ vs 9.7	8.5^a^ vs 10.3

*DOAC* direct oral anticoagulant, *CRNM* clinically relevant non-major, *DOAC* direct oral anticoagulants, *VKA* vitamin K antagonists, *VTE* venous thromboembolism

^a^Statistically significant difference between the two groups

#### Management of VTE in patients with cancer

The major society guidelines including the ACCP, American Society of Clinical Oncology, and the National Comprehensive Cancer Network recommend use of LMWH for treatment of VTE in cancer patients [21, 40, 41]. Treatment with LMWH is continued for the duration of active cancer given that the risk of recurrent VTE can reach an annual risk of 20 % [42]. Five randomized trials have compared therapy with LMWH versus warfarin in cancer patients [43–47]. The details of these trials are outlined in Table 3. Two trials showed a reduction in the rates of recurrent VTE using LMWH with no effect on mortality or bleeding [44, 45], two showed no difference in any outcome [43, 46], and the recently published CATCH trial demonstrated a non-significant reduction in the rate of recurrent VTE and lower risk of CRNMB in those who received LMWH [47].

**Table 3:** Comparison of trials on LMWH versus VKA for treatment of VTE in cancer patients

Trial Name	CANTHANOX	CLOT	MAIN-LITE	ONCENOX	CATCH
Year of Publication [Ref]	2002 [43]	2003 [44]	2006 [45]	2006 [46]	2015 [47]
Design	Open-label	Open-label	Open-label	Open-label	Open-label
Number of Patients	146	676	200	122	900
Treatment Protocol	Enoxaparin 1.5 mg/kg daily	Dalteparin 200 IU/kg once daily for the first month then 150 IU/kg for 5 months	Tinzaparin 175 IU/kg once daily	Enoxaparin 1 mg/kg every 12 h for 5 days then enoxaparin 1 mg/kg or 1.5 mg/kg daily	Tinzaparin 175 IU/kg once daily
Duration of Therapy (months)	3	6	3	6	6
Primary Efficacy Outcome LMWH vs VKA (%)	Combination of major bleeding or recurrent VTE: 10.5 vs 21.1	Recurrent symptomatic VTE: 9^a^ vs 17	Recurrent symptomatic VTE: 7 vs 10	Recurrent symptomatic VTE: enoxaparin 1 mg vs. 1.5 mg vs VKA 6.8 vs 6.3 vs 10.0	Composite of recurrent symptomatic VTE, fatal PE, or incidental VTE: 7.2 vs 10.5
Safety Bleeding Outcomes LMWH vs VKA (%)	Major bleeding: 7 vs 16; Fatal bleeding: 0 vs 8^a^	Major bleeding: 6 vs 4; Any bleeding 14 vs 19	Major bleeding: 7 vs 7; Any bleeding: 27 vs 24	Major bleeding: enoxaparin 1 mg vs. 1.5 mg vs VKA : 6.5 vs 11.1 vs 2.9	Major bleeding: 2.7 vs 2.4 CRNM bleeding: 10.9^a^ vs 15.3

*CRNM* clinically relevant non-major, *DOAC* direct oral anticoagulants, *LMWH* low-molecular weight heparin, *PE* pulmonary embolism, *VKA* vitamin K antagonists, *VTE* venous thromboembolism

^a^Statistically significant difference between the two groups

There are no published randomized trials that a priori have compared DOACs with VKA or LMWH for treatment of VTE in cancer patients. A meta-analysis of the subsets with DVT and cancer totaling 1132 patients in the six trials that compared DOACs versus VKA [15–20] has been published [48]. They found similar rates of VTE recurrence (3.9 versus 6 %; odds ratio [OR] 0.63; 95 % CI, 0.37 – 1.10) and major bleeding (3.2 versus 4.2%; OR 0.77; 95 % CI, 0.41-1.44). Although these trials included cancer patients [15–20], they were typically not receiving active chemotherapy or radiation. The cancer patients included in these trials had usually completed treatment or had a previous history of cancer and are not a true representative of all cancer patients. The Hokusai VTE-cancer randomized open label trial is currently underway and will examine whether edoxaban is non-inferior to LMWH for treatment of VTE in cancer patients [49].

### Extended treatment of venous thromboembolism

Extended anticoagulation can be employed in patients with unprovoked VTE to reduce the risk of recurrent VTE if the benefit/risk ratio favors continuation of anticoagulation while taking into account patient’s risk of bleeding. All DOACs except for edoxaban have been compared with placebo in randomized trials for extended secondary VTE prevention beyond the initial three months of anticoagulation [17, 50, 51]. The details of these trials are compared in Table 4. All trials showed marked superiority of the DOACs over placebo for the prevention of recurrent VTE without significant increase in major bleeding [17,50, 51]. However, compared to the placebo arms, all DOACs had higher rate of CRNMB [17, 50, 51]. Duration of extended anticoagulation was 6 to 12 months in the EINSTEIN [17] and AMPLIFY-Extension [50] studies and 6 months in the RE-SONATE trial [51]. Two doses of apixaban were evaluated in the AMPLIFY-Extension trial and the rate of bleeding was lower for apixaban 2.5 mg twice daily than 5 mg twice daily [50]. A single regimen of rivaroxban (20 mg once daily) and dabigatran (150 mg twice daily) was used in the EINSTEIN and RE-SONATE studies.

**Table 4:** Comparison of extended duration DOAC trials

Trial Name	EINSTEIN-EXTENSION	AMPLIFY-EXT	RE-MEDY	RE-SONATE
Year of Publication [Ref]	2010 [17]	2013 [50]	2013 [51]	2013 [51]
Design	Double-blinded	Double-blinded	Double-blinded	Double-blinded
Comparison Arm	Placebo	Placebo	Warfarin	Placebo
Number of Patients	1197	2486	2866	1353
Treatment Protocol	Rivaroxaban 20 mg once daily	Apixaban 5 mg or 2.5 twice daily	Dabigatran 150 mg twice daily	Dabigatran 150 mg twice daily
Duration of Therapy (months)	6 to12	12	6 to 36	6
Primary Efficacy Outcome DOAC vs VKA or Placebo (%)	Recurrent symptomatic VTE: 1.3^a^ vs 7.1	Recurrent symptomatic VTE or all-cause mortality: 3.8^a^ vs 4.2^a^ vs 11.6	Recurrent symptomatic VTE or related mortality: 1.8^a^ vs 1.3	Recurrent symptomatic VTE or related mortality: 0.4^a^ vs 5.6
Major Bleeding DOAC vs VKA or Placebo (%)	0.7 vs 0	0.2 vs 0.1 vs 0.5	0.9 vs 1.8	0.3 vs 0
Major and CRNM Bleeding DOAC vs VKA or Placebo (%)	6.0^a^ vs 1.2	3.2 vs 4.3 vs 2.7	5.6^a^ vs 10.2	5.3^a^ vs 1.8

*DOAC* direct oral anticoagulant, *CRNM* clinically relevant non-major, *DOAC* direct oral anticoagulants, *VKA* vitamin K antagonists, *VTE* venous thromboembolism

^a^Statistically significant difference between the two groups

Dabigatran is the only DOAC that has been compared with warfarin for extended VTE prevention in the RE-MEDY trial [51]. Dabigatran was non-inferior to warfarin in prevention of recurrent VTE (1.8 versus 1.3 %, hazard ratio [HR] 1.44; 95 % CI, 0.78–2.64) and had a significantly lower rate of major bleeding or CRNMB (HR 0.54; 95 % CI, 0.41–0.71). These results demonstrated that DOACs are effective in secondary VTE prevention with no significant increase in major bleeding. The ACCP guidelines recommend no change in the choice of anticoagulant agent in patients who need extended anticoagulation after the first 3 months of therapy [21]. Given the observed lower bleeding risk, the dose of apixaban may be reduced to 2.5 mg twice daily after the initial treatment.

Aspirin has been also evaluated in secondary VTE prevention in patients with first unprovoked VTE who have completed anticoagulant treatment. In this setting, randomized trials and a meta-analysis reported a 30 % reduction in rates of recurrent VTE compared to placebo or observation [52–55]. The ACCP guidelines suggest that aspirin is an available option in patients with unprovoked VTE that are stopping anticoagulant therapy if there are no contraindications to use of aspirin [21]. However, aspirin is not recommended as an alternative to anticoagulant therapy [21].

### Treatment of VTE in special situations

#### Management of sub-segmental pulmonary embolism

The increase in utilization of a highly sensitive computed tomography pulmonary angiography (CTPA) has led to detection of incidental asymptomatic PE or small sub-segmental PE [56]. Whether or not patients with sub-segmental pulmonary embolism (SSPE) should be anticoagulated is controversial. It is unclear whether the SSPE detected by CTPA are artifacts and therefore false positive [57]. Furthermore, an isolated SSPE likely does not have the same risk of progression or VTE recurrence as a single segmental or lobar PE [57]. There are currently no published randomized trials for treatment of patients with SSPE. Retrospective studies have reported VTE recurrence in only a small number of patients with SSPE and without DVT, who were not anticoagulated. [57, 58]. Details of these retrospective studies are summarized in Table 5 [59–65]. However, another retrospective study showed that patients with SSPE have similar rate of VTE recurrence as patients with larger PE during 3 months of anticoagulation [66]. The ACCP guidelines suggest performing bilateral ultrasounds to exclude proximal DVT before a decision is made not to treat a patient with SSPE [21]. If a DVT is detected then the patient should receive anticoagulation. However, if no proximal DVT is detected the guidelines suggest that the clinician assesses risk factors for VTE recurrence or progression and considers anticoagulation for those with high risk of VTE recurrence (e.g., recent surgery, immobilization, active cancer, previous history of VTE) [21]. Future prospective studies are needed to determine the optimal management strategy for patients with SSPE and no detected proximal DVT.

**Table 5:** Summary of retrospective studies on 3-month follow-up of patients with sub-segmental pulmonary embolism

Study	Musset et al.	Eyer et al.	Donato et al.	Pena et al.	Mehta et al.	Goy et al.	Ghazvinian et al.
Year of Publication [Ref]	2002 [59]	2005 [60]	2010 [61]	2012 [62]	2014 [63]	2015 [64]	2016 [65]
Method of Detection	SDCT	MDCT	MDCT	MDCT	MDCT	MDCT	V/P SPECT
Number of Patient with Positive CTPA	360	499	1463	724	NA^b^	550	NA^b^
Number of Patients with SSPE n/N (%)	12 (3.3)	67 (13.4)	93 (6.4)	70 (9.6)	32 (100)	82 (15)	54 (100)
Number of Untreated SSPE (%)	9 (75)	25 (37.3)	22 (22.9)	18 (25.7)	12 (37.5)	39 (47.6)	54 (100)
VTE (%)	0	0	0	0	0	0	4^a^

*CTPA* computed tomography pulmonary angiography, *NA* not applicable, *MDCT* multi-detector computed tomography pulmonary angiography, *SDCT* single-detector computed tomography pulmonary angiography, *SSPE* sub-segmental pulmonary embolism, *V/P SPECT* ventilation/perfusion singe photon emission computed tomography, *VTE* venous thromboembolism

^a^Two patients were diagnosed with a DVT

^b^Only examined patients with SSPE

#### Role of inferior vena cava filter in management of acute venous thromboembolism

Inferior vena cava (IVC) filters are typically used in patients with an acute VTE and an absolute contraindication to anticoagulation (e.g., concurrent active bleeding) [67]. The IVC filter is removed once the bleeding risk is low and anticoagulation is given [14]. In patients with acute VTE already on anticoagulation with no absolute contraindications, studies suggest that there is lack of benefit to use of IVC filters in addition to anticoagulation [68–72]. In the PREPIC 1 trial, 400 patients with proximal DVT were randomized to either anticoagulation alone or anticoagulation plus IVC filter placement [68]. The initial 2-year PREPIC 1 study and a subsequently published 8-year follow-up reported that IVC filter insertion was associated with a reduction in the initial rate of PE, increase in the rate of DVT, and no difference in mortality [68, 69]. The PREPIC 2 trial examined the adjuvant role of IVC filters in patients with PE who received either anticoagulation alone or anticoagulation plus an IVC filter [70]. The filter was removed at 3 months. There was no difference in the rates of recurrent VTE or mortality between the two groups [70]. In addition to lack of benefit, IVC filters are associated with complications including IVC filter thrombosis, DVT, and guide wire entrapment [71, 72]. The ACCP guidelines recommend against the use of IVC filters in patients on anticoagulation for acute VTE [21].

## Conclusions

VTE is a major cause of morbidity and mortality. DOACs are suggested over VKA for acute and long-term treatment of VTE in patients without cancer, as they have been shown to be as effective as VKA in reducing VTE recurrence and associated with significantly less major bleeding. Future studies are needed to assess their safety and efficacy in outpatient treatment of acute VTE. LMWH is the current standard of care for treatment of VTE in cancer patients. Randomized trials are ongoing to examine the non-inferiority of DOACs versus LMWH in cancer patients. Lastly, it is currently unclear whether or not to treat patients with SSPE and no proximal DVT; future prospective studies are needed to examine different management strategies in this patient group.

## Abbreviations

ACCP: American College of Chest Physicians
CI: Confidence interval
CRNM: Clinically relevant non-major
CTEPH: Chronic thromboembolic pulmonary hypertension
CTPA: Computed tomography of the pulmonary angiography
DOAC: Direct oral anticoagulant
DVT: Deep vein thrombosis
IVC: Inferior vena cava
LMWH: Low-molecular weight heparin
PE: Pulmonary embolism
PESI: Pulmonary embolism severity index
SSPE: Sub-segmental pulmonary embolism
VKA: Vitamin K antagonists
VTE: Venous thromboembolism
